# Luminescent Lanthanoid Calixarene Complexes and Materials

**DOI:** 10.3390/ma10121369

**Published:** 2017-11-28

**Authors:** Massimiliano Massi, Mark I. Ogden

**Affiliations:** Curtin Institute for Functional Materials and Interfaces, Department of Chemistry, Curtin University, GPO Box U 1987, Perth 6845, WA, Australia; m.massi@curtin.edu.au

**Keywords:** lanthanoid, calixarene, luminescence

## Abstract

This review aims to provide an overview of recent examples of lanthanoid-calixarene complexes incorporated into light-emitting materials. Background information on the antenna effect and early work on lanthanoid complexes on calixarenes is provided to set the context. Classes of materials discussed include polymers, nanoparticles, and metal clusters.

## 1. Introduction

**Figure 1:**
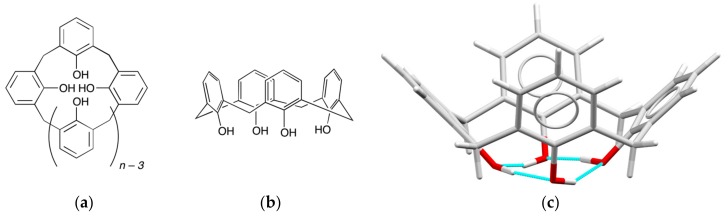
(**a**) Structure of calix[*n*]arene; (**b**) Three dimensional representation of the calix[4]arene in the cone conformation; (**c**) A representation of a structurally characterised calix[4]arene in the cone conformation [10], highlighting the hydrogen bonds (in blue) between the phenol O atoms.

Calixarenes are macrocyclic polyphenols with a wide range of structures and applications [1]. There is a growing library of related macrocyclic systems [2,3], but here we will focus on the systems derived from the reaction of paraformaldehyde with *para*-substituted phenols. The name of these macrocycles derives from the chalice-like structure of the cone conformation of the cyclic tetramer (Figure 1), but they can be isolated in a range of conformations and ring sizes. In the present context, these macrocycles are of interest as ionophores for lanthanoid cations, but it is noted that these macrocycles are excellent receptors for a wide range of metal ions [4]. They are rich in O-donor atoms, readily accessible at significant scales, and can be easily modified, particularly at the phenol O atom, and the *para* position [1]. They also include aromatic rings, which can act as antenna ligands to sensitise the emission of the lanthanoid cations, although antenna moieties can also be added using the macrocycle as a framework [5,6].

Lanthanoid compounds have many applications in materials and life science, exploiting their light-emitting and magnetic properties. Their photophysical properties can be utilised for sensing applications [7,8], biomedical imaging [9], lasers, and Organic Light-Emitting Diodes (OLEDs) [8], to name only a few examples. This review will focus on examples of light-emitting lanthanoid calixarene complexes, setting the context using some of the earliest examples, before moving on to more recent work published since a previous review of this area [6], which is beginning to realise the potential of these systems for light-emitting materials.

## 2. Background

**Figure 2:**
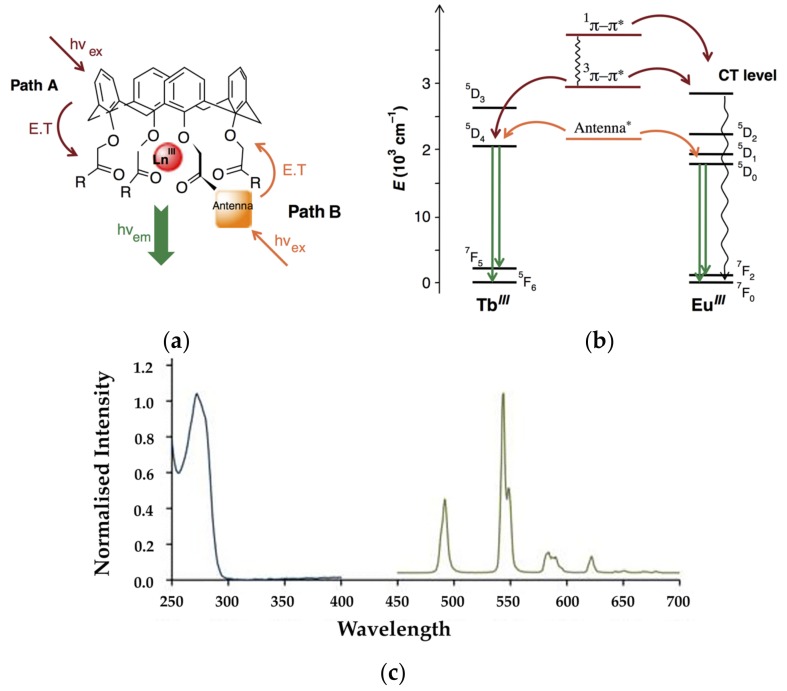
(**a**) Lanthanide emission sensitisation process in a typical lanthanoid calixarene complex. Path A for Ln = Tb and Path B for Ln = Eu. (**b**) Jablonski diagram for the sensitisation of lanthanide luminescence using Path A (in red) or Path B (in orange); (**c**) Typical absorption (blue), and emission spectrum (green) for a terbium calixarene complex showing the line-like emission from the lanthanoid. Figure reproduced with permission of Taylor & Francis Ltd. (www.tandfonline.com) from [14].

A detailed description of the basic photophysics of lanthanoid complexes is beyond the scope of this review, and the reader is referred to the literature for more information [9,11,12,13]. A simple illustration of some of the key concepts is shown in Figure 2 [14]. Effective lanthanoid emission requires sensitisation using an “antenna” ligand to overcome the low absorption cross section of the lanthanoid cation. The lower rim carbonyl substituted calix[4]arene is a very well established ionophoric motif, which allows for the use of the calixarene phenyl rings as the antenna through their ππ* electronic transitions (Path A in Figure 2). Additional antenna moieties can also be introduced at the phenolic rim, allowing for the relative energy levels of the antenna to be adjusted (Path B in Figure 2) to optimize the energy transfer process. The Jablonski diagram in Figure 2b shows simplified energy transfer processes. As is typically assumed, the ligand is excited to its singlet manifold, followed by intersystem crossing to the triplet manifold promoted by the strong spin-orbit coupling of the lanthanoid cation. Energy transfer to the metal centre can then occur, followed by radiative relaxation to the ground state leading to the typical line-like emission from the metal centre due to the inner-core nature of the 4f orbitals (Figure 2c). The excited state of the lanthanoid cation can also undergo non-radiative deactivation, commonly through multiphonon relaxation to coordinated high-energy vibrating bonds, such as O–H, N–H, and C–H. Another complication that could render the antenna effect inefficient is the population of charge transfer states (CT level in Figure 2b), especially in the case of Eu(III) complexes due to the relative stability of the 4f^7^ electronic configuration of Eu(II). Typical examples are represented by Eu(III) complexes where Eu is bound to phenolate [15] or carbonyl O atoms [16] in calixarene receptors. Where the energy level of the calixarene phenyl rings does not lead to efficient energy transfer, the introduction of an alternative antenna using the versatile functionalisation of the calixarene framework may enhance the photophysical properties of the complex by enabling a different pathway (Path B in Figure 2). A full discussion of all of the subtleties and many parameters of a detailed photophysical investigation is beyond the scope of this review. Typical quantities that are routinely measured and reported are the energy of the triplet state of the ligand (through the phosphorescent emission of its corresponding Gd complex), the observed excited state lifetime, and overall quantum yield. It is also possible to measure, or to calculate from the emission spectrum, the value of the radiative lifetime. With this value, it is possible to calculate the intrinsic quantum yield, and hence the sensitisation efficiency of the energy transfer from the ligand to the lanthanoid cation.

## 3. Early Work

**Figure 3:**
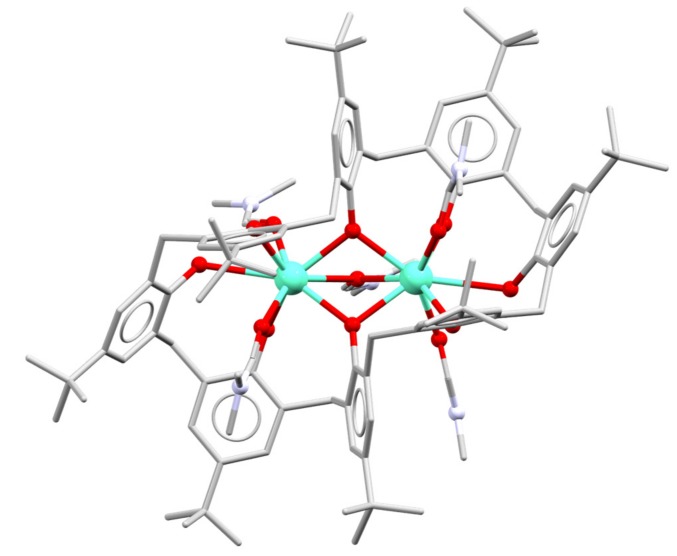
A representation of the bimetallic Eu complex of *p*-t-butylcalix[8]arene [17].

The first lanthanoid calixarene complex was reported in 1987, and was a bimetallic Eu complex of *p*-t-butylcalix[8]arene (Figure 3) [17]. The complex is neutral, so the calixarene retains two phenolic protons. The metal atoms are bridged by two phenolate O-atoms and a solvent Dimethylformamide (DMF) O-atom. The metal atoms are also each coordinated to three phenol O atoms, and two solvent DMF O atoms, giving rise to eight-coordination of each metal centre. Subsequent reports showed that similar complexes could be obtained across the lanthanoid series, as DMF or DMSO solvates [18,19].

The direct binding of a large number of potential antenna units to the lanthanoid cations in these complexes had obvious potential as luminescent species. Photophysical studies of these complexes showed that a low-lying ligand-to-metal charge transfer (LMCT) state gives rise to low quantum efficiency in the Eu complex, due to efficient quenching (or lack of population) of the Eu(^5^D_0_) state. Visually, this LMCT state results in the Eu complex being a distinct orange colour, unlike any other member of the series. The Tb complex, in contrast, is a luminescent species with a relatively high quantum yield, with a detection limit for Tb of 10^−10^ M in DMF solutions [15]. Heteronuclear complexes were also used as model systems to study energy transfer processes between lanthanoid cations, allowing for the distances between metal centres to be estimated [20]. Subsequent studies of the analogous *p*-nitrocalix[8]arene complexes provide an elegant example of how the photophysical properties of lanthanoid complexes can be manipulated by simple changes in the antenna ligand. In fact, it is well known that the energy difference between the triplet state of the antenna and the lanthanoid accepting state play a critical role in establishing the efficiency of the energy transfer process [21]. Here, the introduction of the nitro group shifts the ligand excited states to lower energies. This no longer favours efficient energy transfer from the ligand to Tb(^5^D_4_), but is more favourable for transfer to the lower energy Eu(^5^D_0_) state. The electron withdrawing nitro groups also shift the LMCT state in the Eu complex to higher energy, reducing the efficiency of quenching by that state.

**Figure 4:**
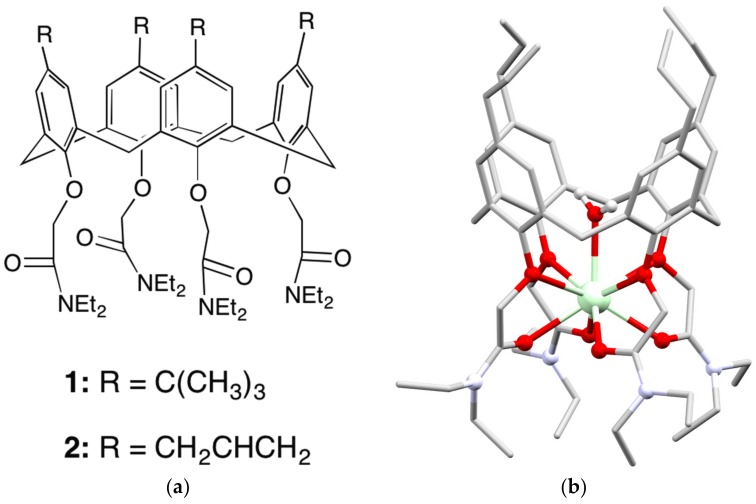
(**a**) The tetraamide substituted calix[4]arenes **1** and **2**; (**b**) A representation of the crystal structure of the [Nd(**2**)(OH_2_)]^3+^ cation [24].

While the parent calixarenes are attractive ionophores for lanthanoids, they have the disadvantage that they do not fully encapsulate the metal ion, allowing for solvent molecules to enter the coordination sphere. Solvent molecules can enable non-radiative relaxation processes via multiphonon relaxation, and hence antenna ligands are more often designed to fully encapsulate the metal. The calix[4]arene is readily alkylated at the phenol O atoms allowing octadentate O donor ligands to be synthesised (Figure 2). One early example was the tetraamide receptor **1** (Figure 4). The Tb complex of this calixarene was studied in solution, and the photophysical properties indicated that one solvent molecule was present in the first coordination sphere [22]. This is achieved by comparing the luminescent lifetimes in H_2_O and D_2_O, as the number of O–H oscillators in the first coordination sphere predictably reduces the lifetime [23]. Interestingly, it was only recently that a similar complex of the *p*-allyl tetraamide **2** was structurally characterised, and this confirmed the presence of a ligated water molecule, at least for Nd and Dy (Figure 4) [24].

**Figure 5:**
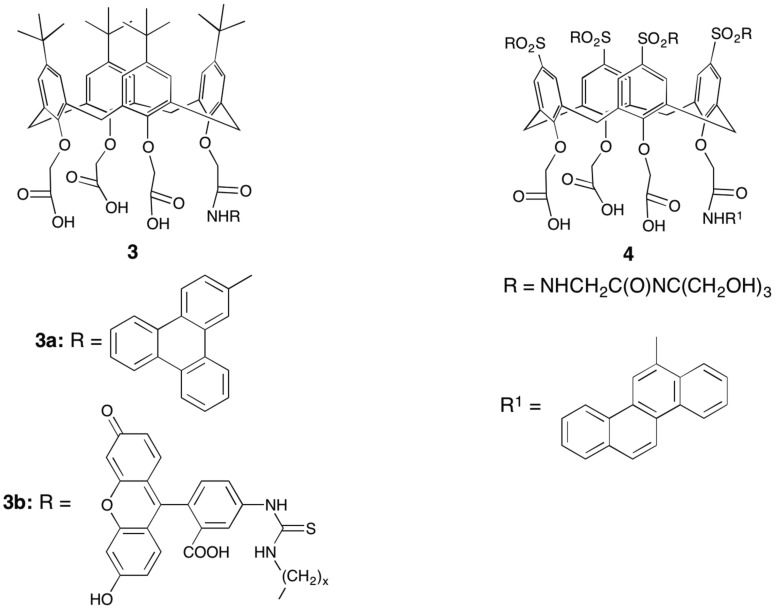
Tetrasubstituted calix[4]arene derivatives that form neutral lanthanoid light emitting complexes.

A broad range of tetrasubstituted calix[4]arene derivatives were subsequently investigated as ionophores for lanthanoids, and their photophysical properties investigated [16,25,26,27]. Elegant examples include the triscarboxylates **3**, **4**, designed to form neutral complexes, with the fourth substituent providing the means of altering the antenna moiety through an amide link (Figure 5) [28]. With a triphenylene antenna chromophore **3a**, both Eu and Tb cations could be sensitised at relatively long excitation wavelengths up to 350 nm, with lifetimes up to 0.73 ms (Eu), or 0.81 ms (Tb) in organic solvents [29]. Attaching a fluorescein antenna group, **3b**, enabled near-infrared emission to be achieved from Nd and Er complexes [30]. The lifetimes for these complexes were much shorter than the visible light emitting systems, as is typical, being 1.23 µs (Nd), and 1.71 µs (Er). Water soluble analogues were made by replacing the *para*-substituent with sulfonamide-linked polyol substituents, **4** (Figure 5) [31]. The chrysene antenna moiety in **4** enabled strong Eu emission in aqueous solution with excitation at 363 nm, achieving lifetime of 0.11 ms. While this lifetime is shorter than achieved in the systems mentioned above, it is notable that this was achieved in the presence of strongly quenching water.

This brief overview of some of the early work in this area highlights the key aspects of calixarenes as antenna ligands for lanthanoid complexes. They are readily functionalised to allow for the tuning of the antenna for specific metal ion, while also effectively encapsulating the metal ion to minimise interaction with solvent molecules. Calixarenes can also be further functionalised to achieve solubility in the desired solvent. We now move on to highlight some of more recent developments with these receptors with an emphasis on systems involving assemblies and materials. For broader coverage of lanthanoid-containing hybrid materials, the reader is referred to other recent reviews [5,6,8,32,33].

## 4. Lanthanoid Calixarene Complexes in Functional Materials

The versatile functionalisation of calixarenes combined with their well established properties, as ionophores have attracted attention recently for the construction of light emitting functional materials. Some recent examples are discussed below.

### 4.1. Polymeric Materials

**Figure 6:**
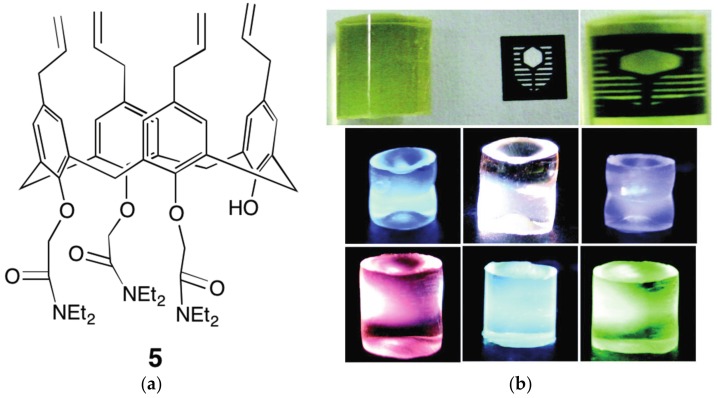
(**a**) The trisamide substituted *p*-allylcalix[4]arene **5**; (**b**) examples of the polymer monoliths showing the transparency of the matric (**top**), and the variation of emitted colours obtained by loading single lanthanoid salts or mixtures. Reproduced by permission of The Royal Society of Chemistry from [35].

The introduction of polymerisable groups onto the calixarene framework is a simple approach to synthesise polymeric functional materials. For example, the tris-amide calix[4]arene **5** was co-polymerised with methyl methacrylate to make cross-linked poly(methyl methacrylate) (PMMA) polymer monoliths containing binding sites for lanthanoid cations [34]. These polymeric materials could be reversibly loaded with the required metal or metal mixtures after synthesis, producing a range of light emitting materials from a single polymeric material, including a white light emitting material (Figure 6) [35]. The aim of using a tris-substituted calix[4]arene was to explore the possibility of sensing applications for ligands binding in the vacant coordination site. Similar polymeric materials have also been reported, incorporating the analogous tetraamide ligand **2** (Figure 4) [24].

Calixarenes have been substituted with polysilsesquioxanes and then chemically linked with poly(4-vinylpyridine) (PVPD) or PMMA, with incorporated lanthanoid cations, to give light emitting materials, with a europium-containing material having the optimal properties amongst those investigated, achieving lifetimes up to 0.71 ms, and a quantum yield of 15% [36]. Such materials potentially offer the combined advantages of organic and inorganic matrices.

Lanthanoid-based coordination polymers have attracted a great deal of attention due to the interesting photophysical and magnetic properties that these cations can add to these systems [33]. Coordination polymers based on calixarenes have been recently reviewed [37]. These are potentially very useful materials, as the calixarene provides a potential binding site beyond those typically found in coordination polymers. Intriguingly, while lanthanoid calixarene coordination polymers are known, and have been used as heterogenous catalysts [38], for example, there appear to be very few examples where the photophysical properties have been studied and exploited [39]. This is an area that has the potential for further investigation, given that reversible photoluminescence switching behaviour has been observed for a Cu(I) calixarene coordination polymer [40].

### 4.2. Nanoparticulate Materials

**Figure 7:**
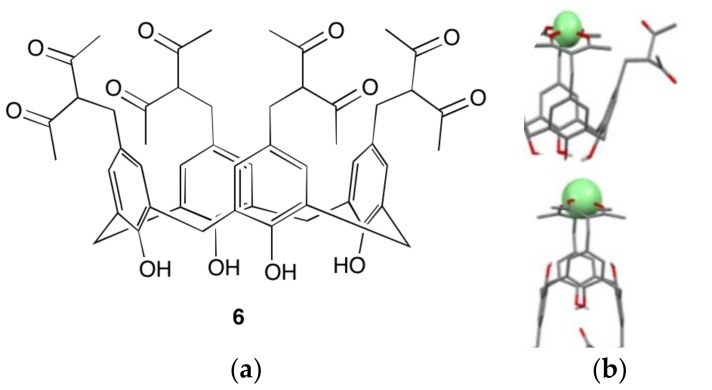
(**a**) The β-diketonate functionalised calix[4]arene **5**; (**b**) A cartoon of the polyelectrolyte (PSS) stabilised nanoparticle with a core of Gd/Tb complexes of **6**, showing interaction with water molecules with Gd, and light with Tb. Reproduced under Creative Commons BY 4.0 from [41].

Nanoparticulate or colloidal materials are of interest for many applications, including biological labelling, drug delivery, and a range of advanced materials. Lanthanoid-calixarene complexes based on the β-diketonate substituted calix[4]arene **6** have recently been used as the functional component of interesting magneto-fluorescent nanoparticles (Figure 7) [41]. These nanoparticles are formed by precipitation upon addition of water to a DMF solution of the metal complex, in the presence of a polyelectrolyte, which stabilises the resulting colloid. By changing the ratio of terbium to gadolinium in the assembly, the magnetic and photophysical properties could be easily adjusted.

While a lanthanoid complex of **6** is yet to be structurally characterised, solution phase studies suggest 1:1 complexes are formed, with a range of coordination modes proposed using computational modelling [42]. As would be expected from earlier studies, this calixarene is an effective antenna ligand for Tb, but much less effective for the near IR emitting Yb. This approach of precipitating highly water-insoluble species to form colloidal suspensions was previously applied to a Tb complex of a resorcinarene cavitand [43], and this could be a very productive methodology across a wide range of hydrophobic lanthanoid calixarene complexes.

**Figure 8:**
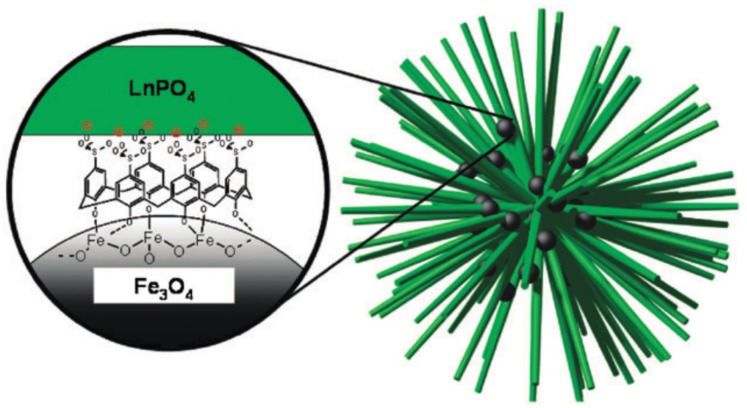
A schematic representation of the nanoball structure of LnPO_4_ nanorods (Ln = La, Eu, Tb, Ce) held together by *p*-sulfonato-calix[6]arene stabilised Fe_3_O_4_ nanoparticles. Reproduced by permission of The Royal Society of Chemistry from [44].

Calixarenes have also been used to link nanoparticles to light-emitting lanthanoid containing particles, as shown in Figure 8. Here the role of the calixarene does not involve the antenna effect, but it stabilises the iron oxide nanoparticles, and provides the link to the lanthanoid phosphate nanorods.

### 4.3. Lanthanoid Clusters

Sitting in the size range between mononuclear complexes, and the nanoparticulate materials described in the previous section, are metal clusters. These polynuclear assemblies are more readily structurally characterised than nanoparticles, and can provide useful information that can inform studies of larger systems, as well as being very useful in their own right. Calixarenes are well known to support metal clusters [45], and even the simple unfunctionalised calixarenes have been found to support multinuclear lanthanoid clusters. The specific cluster that was isolated can depend on very subtle changes in the reaction conditions. For example, reaction of *p*-tert-butylcalix[4]arene with a lanthanoid source and triethylamine in DMF solvent crystallised a dinuclear species [46], whereas the same reaction in a DMF/ROH/CH_3_CN solvent mixture produced hexanuclear clusters [47]. Much of the work in this area focuses on the magnetic properties of the lanthanoid materials [48].

**Figure 9:**
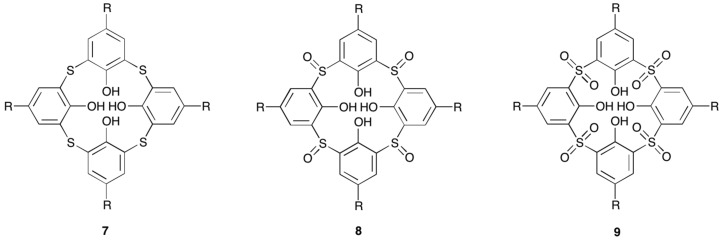
General structures of thiacalix[4]arene, **7**; sulfinylcalix[4]arene, **8**; and, sulfonylcalix[4]arene, **9**.

In the context of lanthanoid (and other metal) clusters, the sulfur-containing thiacalixarenes **7**, and oxidised analogues, the sulfinylcalixarenes **8**, and sulfonylcalixarenes **9**, are particularly interesting, as the moieties linking the phenyl rings also provide potential donor atoms for metal binding (Figure 9). Indeed, the first structurally characterised lanthanoid complex of these ligands was a tetranuclear neodymium cluster sandwiched between two *p*-t-butylthiacalix[4]arene ligands, where the metals were coordinated to both phenol O atoms and the linking S atoms [49]. More recently, *p*-sulfonatothiacalix[4]arene has been found to support a trinuclear lanthanoid cluster in aqueous solution [50].

**Figure 10:**
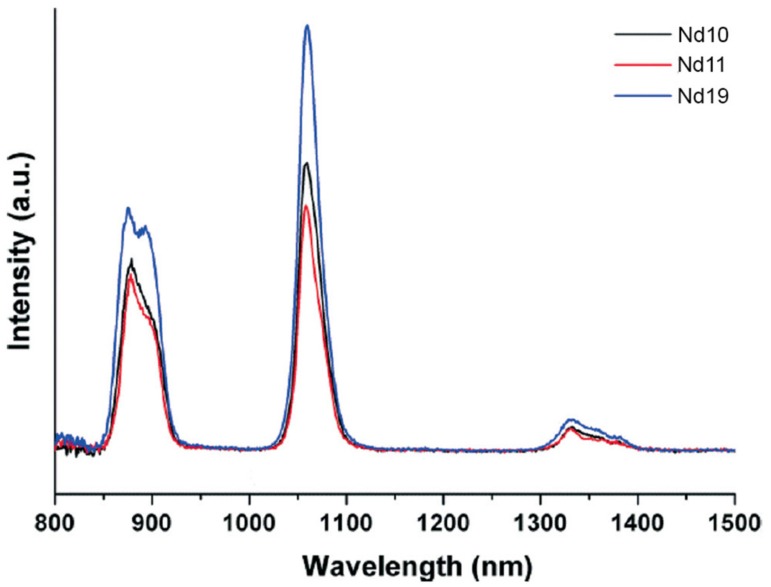
Near Infrared (NIR) luminescence spectra of the Nd_10_, Nd_11_, and Nd_19_ clusters supported by *p*-tert-butylsulfonylcalix[4]arene in the solid state at room temperature. Reproduced by permission of The Royal Society of Chemistry from [51].

The potential these systems have as light emitting materials remains somewhat under-explored. An elegant recent example reports a series of Nd clusters, supported by *p*-tert-butylsulfonylcalix[4]arene and phosphonate co-ligands. Nd_10_, Nd_11_, and Nd_19_ clusters were isolated and structurally characterised, depending on the specific phosphonate coligand used, and the stoichiometry [51]. Upon excitation at 375 nm, the emission spectra of all three clusters exhibited the near IR characteristic bands of the Nd(III) ion (Figure 10).

## 5. Conclusions

This brief overview of the use of lanthanoid calixarene complexes in light emitting materials is intended to highlight the potential areas for future investigation. The use of the wide range of existing water-insoluble complexes to synthesise nanoparticulate materials is one area that seems to have great potential. The ongoing work to better understand the factors that control the formation of metal clusters should benefit the design of light emitting clusters, as well as refining the magnetic properties of such clusters. The ready functionalisation of the calixarene framework, as well as the many other different classes of these macrocycles that were not covered here [52,53], make for endless possibilities that will require careful and systematic design to achieve the best outcomes.
